# Ischemic Preconditioning Potentiates the Protective Effect of Stem Cells through Secretion of Exosomes by Targeting Mecp2 via miR-22

**DOI:** 10.1371/journal.pone.0088685

**Published:** 2014-02-18

**Authors:** Yuliang Feng, Wei Huang, Mashhood Wani, Xiyong Yu, Muhammad Ashraf

**Affiliations:** 1 Medical Research Center of Guangdong General Hospital, Guangdong Academy of Medical Sciences, Guangdong Provincial Cardiovascular Institute, Southern Medical University, Guangzhou, China; 2 Department of Pathology and Laboratory Medicine, University of Cincinnati, Cincinnati, Ohio, United States of America; Northwestern University, United States of America

## Abstract

Mesenchymal stem cells (MSCs) have potential application for the treatment of ischemic heart diseases. Besides differentiation properties, MSCs protect ischemic cardiomyocytes by secretion of paracrine factors. In this study, we found exosomes enriched with miR-22 were secreted by MSCs following ischemic preconditioning (Exo^IPC^) and mobilized to cardiomyocytes where they reduced their apoptosis due to ischemia. Interestingly, by time-lapse imaging, we for the first time captured the dynamic shedding of miR-22 loaded exosomes from cytosol to extracellular space. Furthermore, the anti-apoptotic effect of miR-22 was mediated by direct targeting of methyl CpG binding protein 2 (Mecp2). *In vivo* data showed that delivery of Exo^IPC^ significantly reduced cardiac fibrosis. Our data identified a significant benefit of Exo^IPC^ for the treatment of cardiac diseases by targeting Mecp2 via miR-22.

## Introduction

Ischemic preconditioning has been shown as a potent approach to enhance survival [1] and regeneration of mesenchymal stem cells (MSCs) in an ischemic environment [2]. It has been argued recently that stem cells exert their beneficial effects through the release of beneficial factors [3]. However, the effects of ischemic preconditioning on the paracrine behavior of MSCs are poorly understood.

Exosomes have been reported as key transporters of paracrine factors. Recently, it has been reported that microRNAs, a large family of small non-coding RNAs (22–24 nucleotides long), mediate their cellular effects through exosomes [4].

We hypothesized that exosomes secreted by MSCs communicate with cardiomyocytes for the transfer of miRNAs to improve cardiac function after myocardial infarction via regulation of the expression of specific genes.

In this study, we employed microRNA microarray to profile the miRNAs in the exosomes of MSCs subjected to ischemic preconditioning (Exo^IPC^) and focused on their effects on ischemic cell survival. We confirmed the transfer and movement of miRNAs by co-culture and time-lapse imaging. Lastly, we demonstrated the effects of Exo^IPC^ in infarcted myocardium.

## Method

### Ethics Statement

All animal experimental procedures conform to the Guide for the Care and Use of Laboratory Animals published by the US National Institutes of Health (NIH Publication #85-23, revised 1996) and were conducted according to a protocol approved by the Institutional Animal Care and Use Committee, University of Cincinnati.

### Isolation and culture of MSCs

We isolated and purified bone marrow-derived MSCs from C57Black-6 mice by flushing the cavity of femurs as described previously [5]. Cells were cultured using serum-free medium (STEMPRO® MSC SFM, Invitrogen).

### Ischemic Preconditioning of MSCs

MSCs were seeded at 5×10^5^ cells/60-mm cell culture dish. The cells were starved overnight of glucose before preconditioning. For ischemic preconditioning, the cells were subjected to repeated cycles of anoxia (30 min) with intermittent reoxygenation (10 min) for two cycles in an anoxic chamber (Forma-1025 Anaerobic System).

### Exosome Isolation

The isolation procedures for exosomes were modified from a previous protocol [6]. MSCs were washed with PBS 3 times and subcultured in serum free medium (STEMPRO® MSC SFM). Residual FBS in culture media was pre-cleared by ultracentrifugation (100,000 *g*, 3 hrs, 4°C). Supernatant collected from MSC cultured dishes was filtered through 0.3 µm pore filters (Millipore) as previously described [4] followed by ultracentrifugation (20,000 *g*, 20 minutes) and incubation with an exosome precipitation solution. Exosomes were then harvested after ultracentrifugation at 100,000 g for 70 minutes as previously described or as per manufacturer's protocol. Briefly, culture medium was collected and centrifuged at 3000× *g* for 15 minutes. Then the supernatant was transferred to a sterile vessel and the appropriate volume of ExoQuick Exosome Precipitation Solution (SBI) was added, followed by refrigeration overnight (16 hours). After refrigeration, ExoQuick/supernatant mixture was centrifuged at 1500× *g* for 30 minutes and supernatant was aspirated and the vessel was spun down again to remove residual ExoQuick solution by centrifugation at 1500× *g* for 5 minutes.

### Exosomal RNA isolation and microRNA profiling

Total RNA was extracted with *mir*VanaTM miR isolation kit. C. elegans miR-39 (cel-miR-39) was used as an internal control due to its lack of sequence homology to mouse miRs and absence of empiric hybridization to mouse miR probes on miR microarrays [7]. qRT-PCR for miRs of interest (SYBR Green) in exosomes was performed on Biorad. All PCR reactions were run in triplicate, and miR expression relative to U6 or cel-miR-39 was calculated using the 2^−ΔΔCt^ method.

### Time lapse confocal imaging

Time lapse confocal imaging was performed using Zeiss LSM 710 Confocal Microscope (Live Microscopy Core, University of Cincinnati). MSCs were transduced with lentivirus pCT-CD63-RFP overexpressing fusion protein CD63-RFP for 24 hr, washed with fresh medium 3 times (5 min/each). After washes, the MSCs were transfected with miR-22 mimics (3′ fluorescein labeled) for 24 hr washed with fresh STEMPRO® MSC SFM medium (mixed with RNAase) for 3 times (5 min/each) and added fresh STEMPRO® MSC SFM in the end. 35 mm culture dishes with MSCs were placed into the incubation chamber maintained at 37°C and 5% CO_2_. Multi-channel time-series images were captured every 30 seconds using either Plan-Apochromat 20×/0.8 dry objective or LDC-Apochromat 40×/1.0 water immersion objective. Green channel was excited with 488 nm laser (emission 495–550), red channel excited with 560 nm laser (emission 565–665), and a transmitted light channel in white.

### TUNEL

Apoptosis was detected by TUNEL according to instructions of the manufacturer (Roche Applied Science). For quantification, the numbers of TUNEL^+^ cells were counted in at least five randomly selected high power fields (magnification, ×200) with three independent samples by one of the authors who had no knowledge of the treatment.

### Western blot Analysis

Western blot analysis was performed as described in detail [8]. Briefly, total proteins were extracted with lysis buffer (50 mM Tris-HCl, pH 7.6, 120 mM NaCl, 0.5% NP-40, 1 mM EDTA, 0.1 mM PMSF, 2 µg/ml Leupeptin, 2 µg/ml Aprotinin). Proteins were separated by NuPAGE® Novex® 4–12% Bis-Tris Gels (Invitrogen) and transferred onto nitrocellulose membrane (Biorad). Membranes were incubated in Tris-buffered saline with 5% nonfat milk containing antibodies against Mecp2 (courtesy of Prof. Michael E. Greenberg, Harvard University) at 4°C overnight. After washing, the membranes were incubated with horseradish peroxidase-conjugated secondary antibodies, and visualized using the ECL chemiluminescence system (Amersham) or SuperSignal West Femto Chemiluminescent Substrate (Pierce).

### Transfection of miR mimic and inhibitor and luciferase reporter

For each transfection in 1 well of a 6-well plate, miR-22 mimic (100 nM, Thermo) or inhibitor (50 nM, Exiqon) was used. For luciferase reporter transfection, 100 ng of plasmid was transfected in 1 well of a 6-well plate. Luciferase assays were performed as described previously with the Dual-Luciferase reporter assay system (Promega) [1].

### Mouse MI model

A myocardial infarction (MI) model was established in C57BL/6J mice (8–10 week old). The mice were given general anesthesia with 1–2% isoflurane. Animals were mechanically ventilated using a rodent ventilator (Model 683, Harvard Apparatus, South Natick, MA) connected to an endotracheal tube. The heart was exposed by a left side limited thoracotomy and the left anterior descending artery (LAD) was ligated with a 6-0 polyester suture 1 mm from tip of the normally positioned left auricle.

### *In vivo* delivery of exosomes (Exo^non-IPC^, Exo^IPC^, Exo^IPC+miR-22 Inhibitor^), miR-22 mimic, Mecp2 siRNA into ischemic hearts

With the chest open, synthetic oligonucleotides pretreated with 15 µl Dharmafect Duo (Thermo) were injected into the myocardium through an insulin syringe with a 29-gauge needle (BD). The dosages used per mouse were 80 ng miR-22 mimic, 1 nmol Mecp2 siRNAs On-TARGETplus SMART pool (a pool of four target-specific 20–25 nt siRNAs; Thermo). One µg exosomes (Exo^non-IPC^, Exo^IPC^, Exo^IPC+miR-22 Inhibitor^) were injected along the border between infarct zone and normal myocardium after LAD.

### Analysis of left ventricular (LV) fibrotic area

Heart specimens (n = 8 for each group) from mice 4 weeks after the exosomal injection were fixed with 10% (v/v) buffered formaldehyde, dehydrated in graded ethanol series, embedded in paraffin, and cut into 5-µm thick sections. The slices stained with Masson's Trichrome were used to quantify fibrosis in the left ventricle in various treatment groups. An Olympus BX41 microscope equipped with CCD (Magna-Fire TM, Olympus) camera captured LV area images on each slide. LV fibrotic area and total LV area of each image were measured using the Image-Pro-Plus (Media Cybernetics Inc., Carlsbad, CA, USA), and the fibrotic area was reported as a percentage of the total LV area.

### Statistical Analysis

All experiments were repeated at least three times. Data were represented as mean±SEM. Significance of differences between two groups was tested by Student's t test or ANOVA. A P value less than 0.05 or 0.01 was regarded as significant.

## Results

### microRNA profiling in exosomes released from MSCs after ischemic preconditioning

**Figure 1:**
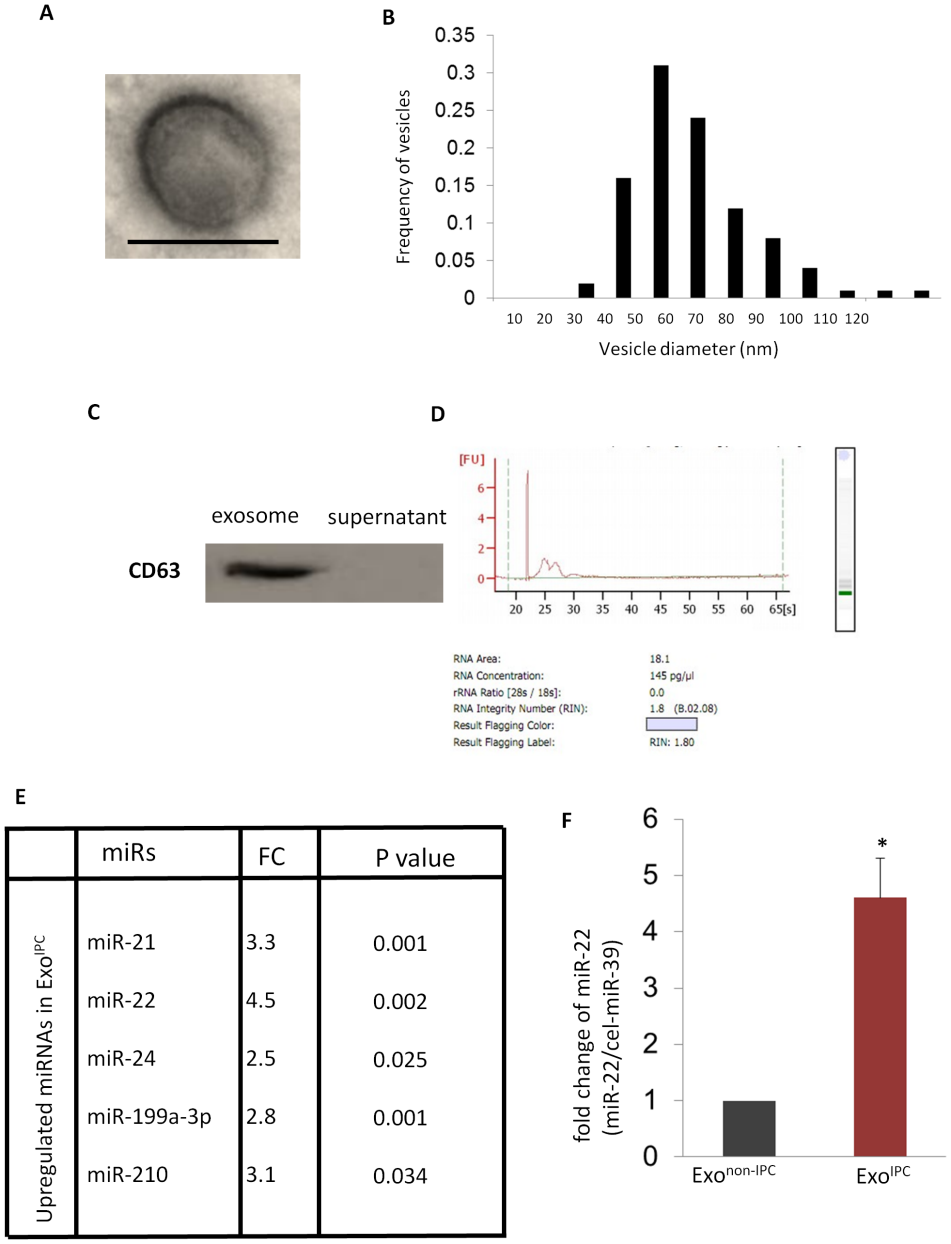
miR-22 is upregulated in Exo^IPC^. **A.** Electron microscopy was used to image the structure of an exosome, which was approximately 100 = 100 nm. **B.** Histogram showing exosome size distribution. X values: Vesicle diameter (nm); Y value: frequency of vesicles. **C.** Equal amounts of proteins from exosomes and culture medium were analyzed by western blotting for the exosome-enriched protein, CD63. **D.** RNA analysis by bioanalyzer showed no ribosomal RNA 28 s and 18 s presence. **E.** microRNA microarray data showing upregulation of miRs in Exo^IPC^ as compared to Exo^non-IPC^. Exo^IPC^ exosomes from MSCs following ischemic preconditioning. Exo^non-IPC^: exosomes from MSCs without ischemic preconditioning. FC: fold change (Exo^IPC^ vs. Exo^non-IPC^). **F.** qPCR was performed to confirm the enrichment of miR-22 in exosome^IPC^ as compared to exosome^non-IPC^. cel-miR-39 was used as invariant control. (*) denotes *P*<0.05 for significant difference (n = 3). Exo^IPC^ exosomes from MSCs following ischemic preconditioning.

To determine the miRNA repertoires of exosomes secreted by MSCs after ischemic preconditioning, we isolated exosomes from cell supernatants of MSCs with and without ischemic preconditioning. Exosomes were isolated by ExoQuick-TC™ precipitation solution and the purity of exosomal fractions was assessed by transmission electron microscopy, western blotting and bioanalyzer for RNA. Transmission electron microscopy showed round-shaped membranous vesicles measuring from 30 to 120 nm in diameter with a mean size of 57.4 nm (**Fig. 1B**). These descriptions are consistent with previous reports [9]. Western blot of extracellular particles isolated from the culture medium from MSCs cultured dishes had immunoreactivity with a specific antibody against CD63, one of the key exosomal membrane proteins (**Fig. 1C**). Moreover, bioanalyzer results showed that the RNAs isolated from the culture medium of MSC dishes contained no 28s/18s ribosomal RNA (**Fig. 1D**), indicating the cellular contamination was minimal. microRNA microarray analysis of exosomes showed that the number and expression level of miRs were different in exosomes secreted from MSCs subjected to ischemic preconditioning (Exo^IPC^) as compared to exosomes secreted from non- preconditioned MSCs (Exo^non-IPC^) (**Fig. 1E**). In particular, we found that miR-22 was highly up-regulated in Exo^IPC^. We next validated and confirmed the higher levels of miR-22 in 4 additional Exo^IPC^ samples (**Fig. 1F**).

### Transfer of secreted miR-22 to cardiomyocytes

**Figure 2:**
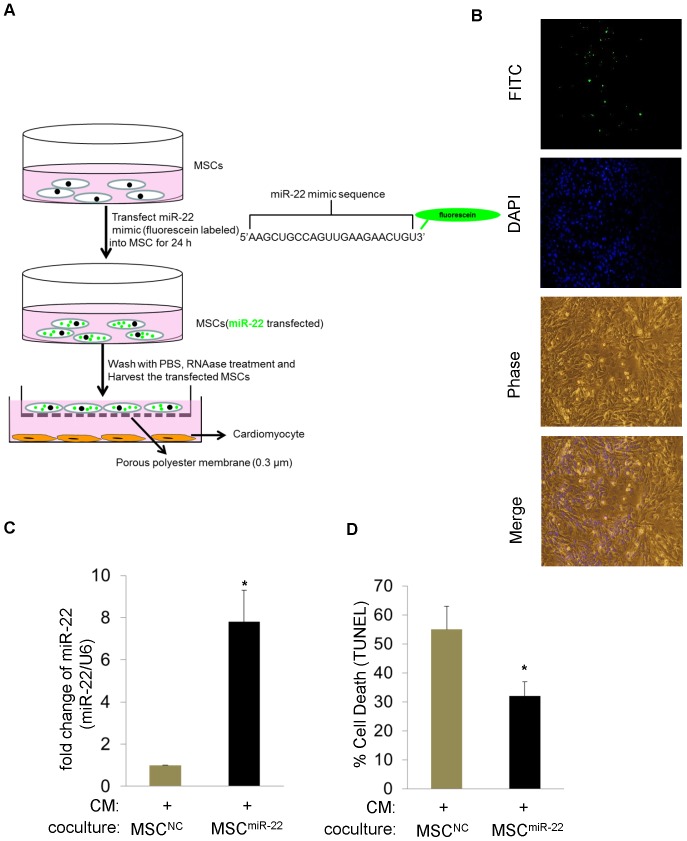
miR-22 transfers from MSCs to cardiomyocytes. **A.** A diagrammatic representation of experimental design for visualizing the transfer of miR-22 from MSCs into cardiomyocytes in a co-culture system. **B.** Fluorescent microscopy showed the existence of miR-22 (Green) in cardiomyocytes after 24 hr co-culture with MSCs. **C.** qPCR analysis showed the significant upregulation of miR-22 in cardiomyocytes co-cultured with MSCs^miR-22^ as compared to MSCs^NC^ (MSCs^miR-^22; MSCs transfected with miR-22 mimic; MSCs^NC^ transfected with negative control of microRNA mimic). (*) denotes *P*<0.05 for significant difference (n = 3). **D.** TUNEL assay showed reduced apoptosis in cardiomyocytes (co-cultured with MSCs^miR-^22) as compared to control. (*) denotes *P*<0.05 for significant difference (n = 3).

To determine whether miR-22 secreted from MSCs can transfer to cardiomyocytes in a non-contact manner, first, we transfected MSCs with a miR-22 mimic that was labeled with green fluorescein (MSC^miR-22^) and then washed the MSCs with RNAse containing DMEM to degrade the residual miR-22 mimic on the MSCs. Figure S1 has shown RNase was effective. Then we employed a co-culture system of MSCs with neonatal cardiomyocytes in which the cells were separated by a membrane of 0.3 µm pore size to prevent direct cell contact or transfer of larger vesicles (**Fig. 2A**). Fluorescent imaging showed that after co-culture for 24 h, the green miR-22 was mainly concentrated in cardiomyocytes (**Fig. 2B**). In parallel, real-time PCR demonstrated that the expression of miR-22 was dramatically upregulated in cardiomyocytes co-cultured with MSCs. Moreover, these cardiomyocytes were more resistant to ischemic stress (**Fig. 2C**). These experiments demonstrate that miR-22 can mobilize from MSCs to cardiomyocytes.

**Figure 3:**
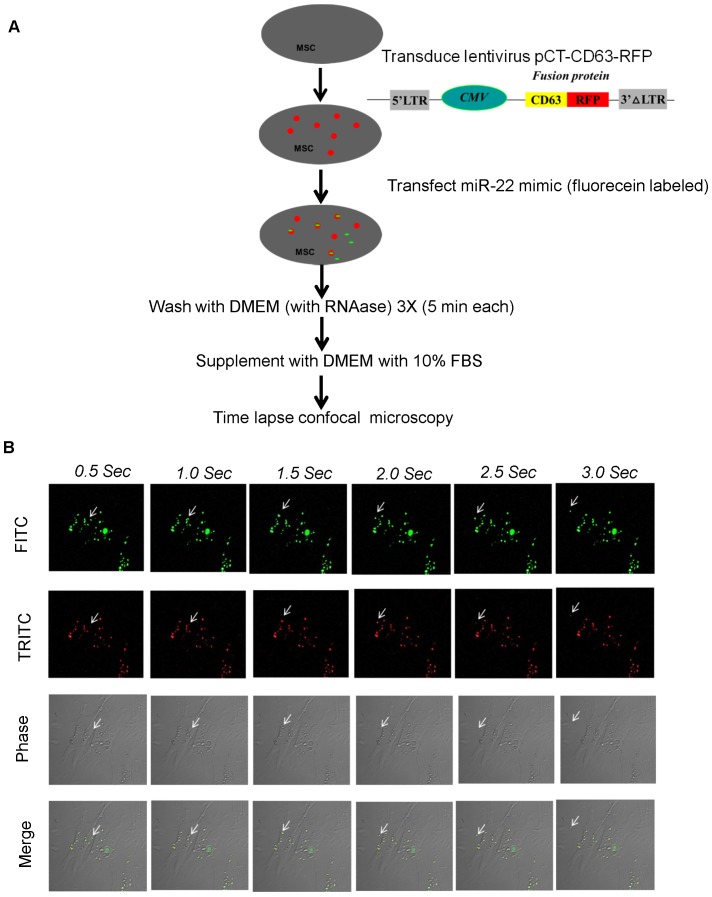
Time-lapse imaging for the transfer of exosomal miR-22. **A.** Flow chart of time-lapse confocal imaging experiment. **B.** Live cell imaging of MSCs co-transfected with lentivirus overexpressing fusion protein CD63-RFP (red) and miR-22 labeled with fluorescein (green).

In order to capture the dynamic shedding of miR-22 from MSC, we employed time-lapse confocal imaging. First, we transduced lentivirus pCT-CD63-RFP in MSCs to label exosomes by overexpressing the fusion protein CD63-RFP. Then we transfected fluorescein labeled miR-22. Colocalization of red fusion protein CD63-RFP and green miR-22 indicated that miR-22 transferred to exosomes (**Fig. 3A**). Very interestingly, we captured the dynamic shedding course of miR-22 loaded exosomes from cytosol into extracellular space (**Fig. 3B** **and Video S1**). This is the first report to visualize microRNA shedding via exosomes.

### miR-22 targets Mecp2

**Figure 4:**
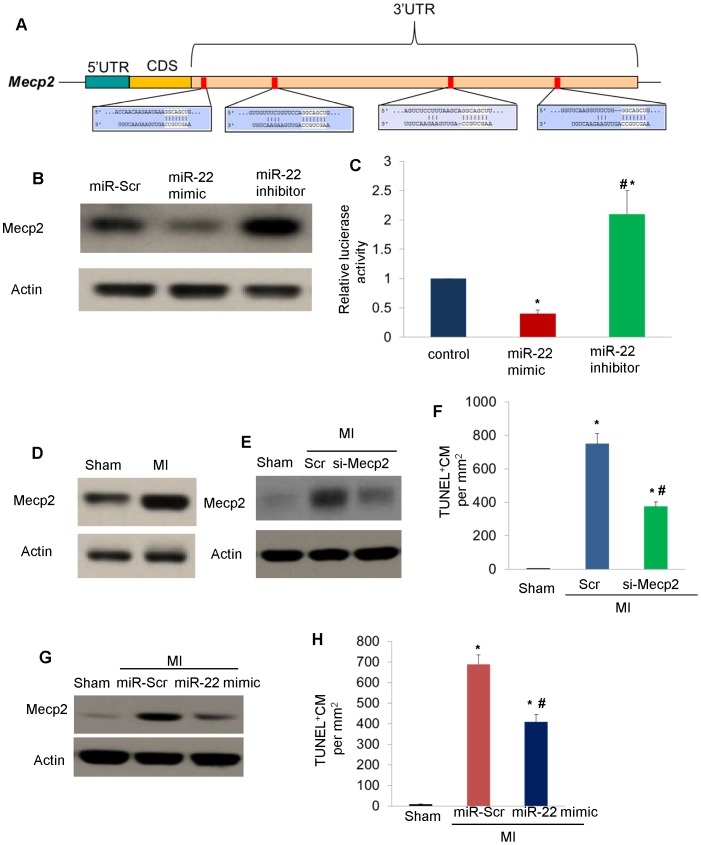
miR-22 targets Mecp2. **A.** Target scan showed that Mecp2 is predicted target of miR-22 with 4 potential binding sites on its 3′UTR. **B.** Western blot was performed in MSCs post transfection of microRNA scramble (miR-Scr), miR-22 mimic (100 nM) and miR-22 LNA inhibitor (50 nM) in MSCs. **C.** Luciferase activity was employed in MSCs post transfection of microRNA scramble (miR-Scr), miR-22 mimic (100 nM) and miR-22 LNA inhibitor (50 nM). (*) denotes *P*<0.05 vs. control for significant difference (n = 3). (#) denotes *P*<0.05 vs. miR-22 mimic for significant difference (n = 3). **D.** Western blot showing upregulation of Mecp2 in infarcted hearts. **E.** Western blot showing successful knockdown of Mecp2 in infarcted hearts with siRNA (si-Mecp2) **F.** TUNEL assay showing reduction of apoptosis in ischemic cardiomyocytes by si-Mecp2. (*) denotes *P*<0.05 vs. control for significant difference (n = 3 for each group). (#) denotes *P*<0.05 vs. miR-22 mimic for significant difference (n = 3). **G.** Western blot showing downregulation of Mecp2 in infarcted hearts by miR-22 mimic. **H.** TUNEL assay showing reduction of apoptosis in ischemic cardiomyocytes by miR-22 mimic. (*) denotes *P*<0.05 vs. control for significant difference. (#) denotes *P*<0.05 vs. miR-22 mimic for significant difference (n = 3 for each group).

In order to investigate the direct downstream effectors/targets by which miR-22 exerts its function, we used computational algorithms to predict potential miR-24 target genes. Computational analysis showed Mecp2 is a potential target of miR-22 by binding on the 3′UTR (**Fig. 4A**). Therefore, we interrogated the relationship of miR-22 and Mecp2 in cardiomyocytes. As shown in (**Fig. 4B**), transfection of miR-22 mimic downregulated Mecp2 in cardiomyocytes while transfection of miR-22 inhibitor upregulated Mecp2 in cardiomyocytes. Moreover, luciferase reporter assay in which the luciferase reporter gene was fused to the wild-type Mecp2 3′UTR showed that luciferase activity in MSCs transfected with miR-22 mimic was downregulated while it was upregulated in MSCs transfected with miR-22 inhibitor (**Fig. 4C**). From these data, we concluded that the miR-22 targets Mecp2. Moreover, we found Mecp2 was upregulated in infarcted hearts as shown in (**Fig. 4D**). Furthermore, knockdown of Mecp2 by specific siRNAs in infarcted hearts (**Fig. 4E**) reduced apoptosis in the ischemic myocardium (**Fig. 4F**). Similarly, delivery of miR-22 mimic significantly downregulated Mecp2 (**Fig. 4G**) and ameliorated apoptosis in ischemic myocardium (**Fig. 4H**). Taken together, these data showed Mecp2 is a functional target of miR-22.

### In vivo delivery of miR-22 loaded exosomes ameliorated fibrosis post myocardial infarction

**Figure 5:**
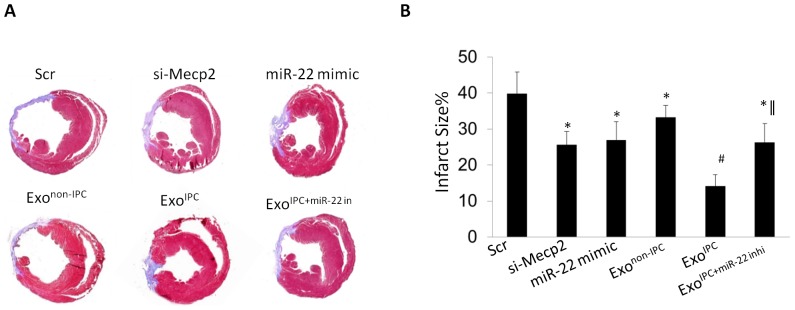
Exo^IPC^ ameliorated cardiac fibrosis. **A**. Masson trichrome staining to determine the infarct size in various groups. **B**. Quantification of infarct size in various groups. (*) denotes *P*<0.05 vs. Scr for significant difference. (#) denotes *P*<0.05 vs. si-Mecp2, miR-22 mimic, Exo^non-IPC^, Exo^IPC+miR-22 inhi^ for significant difference (n = 8). (∥) denotes *P*<0.01 vs. Exo^non-IPC^ for significant difference (n = 8 for each group).

In order to confirm the role of miR-22 loaded exosomes in heart repair, we injected scramble, si-Mecp2, miR-22 mimic, and Exo^IPC^, Exo^non-IPC^, Exo^IPC+ miR-22 inhi^ into infarcted hearts. Masson trichrome staining showed delivery of si-Mecp2 and miR-22 mimic significantly reduced cardiac fibrosis as compared to scramble. Delivery of miR-22 loaded exosomes resulted in significant reduction of fibrotic area as compared to Exo^non-IPC^, si-Mecp2, miR-22 mimic. However, delivery of exosomes from MSCs after inhibition of miR-22 prior to IPC (Exo^IPC+ miR-22 inhi^) mitigated anti-fibrotic effects (**Fig. 5A and B**). Taken together, miR-22 is a critical factor in mediating the cardioprotective effect of Exo^IPC^ on cardiac remodeling post myocardial infarction via targeting Mecp2.

## Discussion

MSCs transplantation has been reported to be an effective intervention for ischemic cardiomyopathy [10], [11] and it has also been shown to attenuate cardiac remodeling and reduce apoptosis through paracrine factors [12], [13]. The current study exploits the benefits of preconditioning through the secretion of exosomes from MSCs which were responsible for delivery of bioactive molecules.

Although many reports showed that miRNAs loaded exosomes could be secreted from the cells [14], [15], these studies represented indirect role of exosomes in molecule delivery but did not reporte any direct evidence of the dynamic shedding course of exosomes into extracellular space. Using state-of-art time-lapse confocal imaging, we successfully captured the dynamic course of miRNA loaded exosome release from the MSCs. This is the first report to provide the direct evidence of miRNA loaded exosomes shedding from the stem cells.

Our data showed that delivery of miR-22 reduced apoptosis in ischemic cardiomyocytes, ameliorated fibrosis and improved cardiac function post-myocardial infarction, which is partially in agreement with a recent study [16] showing that the inhibition of miR-22 affected the survival of cardiomyocytes after isoproterenol treatment. Although this study in contrast to a previous report [17], which showed that miR-22 is a regulator in cardiac hypertrophy (TAC or ISO model), demonstrated that miR-22 is critical for the cardioprotection of myocardial infarction after treatment with ischemic preconditioned MSCs, these discoveries suggest different roles for miR-22 in different pathophysiological processes or conditions.

Moreover, we found that miR-22 protected ischemic hearts at least partially by targeting Mecp2, which was previously reported to be upregulated in the ischemic heart [18]. We demonstrated that the treatment of infarcted hearts with Exo^IPC^ resulted in a better cardiac outcome as compared to treatment with the miR-22 mimic or Mecp2. Furthermore, the inhibition of miR-22 prior to IPC partially mitigated the cardioprotective effect of Exo^IPC^, suggesting a possible role of other cardioprotective miRs or factors (such as miR-21, miR-199a-3p, and miR-210) cannot be underestimated.

Elevated Mecp2 expression has been reported in cardiac and skeletal abnormalities during development [19] and our siRNA study showed that inhibition of Mecp2 could protect ischemic hearts. However, as an epigenetic regulator, the specific targets of Mecp2 in these pathologic processes are still unknown. A potent ChIP (chromatin immunoprecipitation) grade antibody for ChIP-seq study may answer this question.

Taken together, the identification of exosomes released from MSCs as a shuttle that carries miR-22 to ischemic cardiomyocytes allows us to assign a role to exogenous MSCs in cardiac recovery after myocardial infarction.

Moreover, our discovery into the role of exosomes adds another dimension to the preconditioning paradigm and leads to further understanding into how we can exploit its application for the treatment of cardiovascular diseases with the use of stem cells in the future.

## Supporting Information

Video S1 (AVI)

Figure S1 (DOCX)

## References

[1] Kim HW, Haider HK, Jiang S, Ashraf M (2009) Ischemic preconditioning augments survival of stem cells via miR-210 expression by targeting caspase-8-associated protein 2. J Biol Chem 284: 33161–33168. 19721136 10.1074/jbc.M109.020925 PMC2785158

[2] Leroux L, Descamps B, Tojais NF, Seguy B, Oses P, et al (2010) Hypoxia preconditioned mesenchymal stem cells improve vascular and skeletal muscle fiber regeneration after ischemia through a Wnt4-dependent pathway. Mol Ther 18: 1545–1552. 20551912 10.1038/mt.2010.108 PMC2927059

[3] Ranganath SH, Levy O, Inamdar MS, Karp JM (2012) Harnessing the mesenchymal stem cell secretome for the treatment of cardiovascular disease. Cell Stem Cell 10: 244–258. 22385653 10.1016/j.stem.2012.02.005 PMC3294273

[4] Valadi H, Ekstrom K, Bossios A, Sjostrand M, Lee JJ, et al (2007) Exosome-mediated transfer of mRNAs and microRNAs is a novel mechanism of genetic exchange between cells. Nat Cell Biol 9: 654–659. 17486113 10.1038/ncb1596

[5] Buccini S, Haider KH, Ahmed RP, Jiang S, Ashraf M (2012) Cardiac progenitors derived from reprogrammed mesenchymal stem cells contribute to angiomyogenic repair of the infarcted heart. Basic Res Cardiol 107: 301. 23076626 10.1007/s00395-012-0301-5 PMC3505546

[6] Roccaro AM, Sacco A, Maiso P, Azab AK, Tai YT, et al (2013) BM mesenchymal stromal cell-derived exosomes facilitate multiple myeloma progression. J Clin Invest 123: 1542–1555. 23454749 10.1172/JCI66517 PMC3613927

[7] Kroh EM, Parkin RK, Mitchell PS, Tewari M (2010) Analysis of circulating microRNA biomarkers in plasma and serum using quantitative reverse transcription-PCR (qRT-PCR). Methods 50: 298–301. 20146939 10.1016/j.ymeth.2010.01.032 PMC4186708

[8] Lu G, Haider HK, Jiang S, Ashraf M (2009) Sca-1+ stem cell survival and engraftment in the infarcted heart: dual role for preconditioning-induced connexin-43. Circulation 119: 2587–2596. 19414636 10.1161/CIRCULATIONAHA.108.827691 PMC2839162

[9] Thery C, Amigorena S, Raposo G, Clayton A (2006) Isolation and characterization of exosomes from cell culture supernatants and biological fluids. Curr Protoc Cell Biol Chapter 3: 3–22. 10.1002/0471143030.cb0322s30 18228490

[10] Gnecchi M, Danieli P, Cervio E (2012) Mesenchymal stem cell therapy for heart disease. Vascul Pharmacol 57: 48–55. 22521741 10.1016/j.vph.2012.04.002

[11] Pasha Z, Wang Y, Shiekh R, Zhang D, Zhao T, et al (2008) Preconditioning enhances cell survival and differentiation of stem cells during transplantation in infarcted myocardium. Cardiovasc Res 77 1: 134–42. 18006467 10.1093/cvr/cvm025

[12] Uemura R, Xu M, Ahmad N, Ashraf M (2006) Bone marrow stem cells prevent left ventricular remodeling of ischemic heart through paracrine signaling. Circ Res 98: 1414–1421. 16690882 10.1161/01.RES.0000225952.61196.39

[13] Gnecchi M, He H, Liang OD, Melo LG, Morello F, et al (2005) Paracrine action accounts for marked protection of ischemic heart by Akt-modified mesenchymal stem cells. Nat Med 11: 367–368. 15812508 10.1038/nm0405-367

[14] Hergenreider E, Heydt S, Treguer K, Boettger T, Horrevoets AJ, et al (2012) Atheroprotective communication between endothelial cells and smooth muscle cells through miRNAs. Nat Cell Biol 14: 249–256. 22327366 10.1038/ncb2441

[15] Mittelbrunn M, Gutierrez-Vazquez C, Villarroya-Beltri C, Gonzalez S, Sanchez-Cabo F, et al (2011) Unidirectional transfer of microRNA-loaded exosomes from T cells to antigen-presenting cells. Nat Commun 2: 282. 21505438 10.1038/ncomms1285 PMC3104548

[16] Huang ZP, Chen J, Seok HY, Zhang Z, Kataoka M, et al (2013) MicroRNA-22 regulates cardiac hypertrophy and remodeling in response to stress. Circ Res 112: 1234–1243. 23524588 10.1161/CIRCRESAHA.112.300682 PMC3720677

[17] Gurha P, Abreu-Goodger C, Wang T, Ramirez MO, Drumond AL, et al (2012) Targeted deletion of microRNA-22 promotes stress-induced cardiac dilation and contractile dysfunction. Circulation 125: 2751–2761. 22570371 10.1161/CIRCULATIONAHA.111.044354 PMC3503489

[18] Katare R, Riu F, Mitchell K, Gubernator M, Campagnolo P, et al (2011) Transplantation of human pericyte progenitor cells improves the repair of infarcted heart through activation of an angiogenic program involving micro-RNA-132. Circ Res 109: 894–906. 21868695 10.1161/CIRCRESAHA.111.251546 PMC3623091

[19] Alvarez-Saavedra M, Carrasco L, Sura-Trueba S, Demarchi AV, Walz K, et al (2010) Elevated expression of MeCP2 in cardiac and skeletal tissues is detrimental for normal development. Hum Mol Genet 19: 2177–2190. 20203171 10.1093/hmg/ddq096

