# Supplementary material for: Ischemic Preconditioning Potentiates the Protective Effect of Stem Cells through Secretion of Exosomes by Targeting Mecp2 via miR-22
Source: PLoS One. 2014 Feb 18;9(2):e88685. doi: 10.1371/journal.pone.0088685 (PMC3928277; doi:10.1371/journal.pone.0088685)
Supplement: Figure S1 — (DOCX) [file pone.0088685.s002.docx]

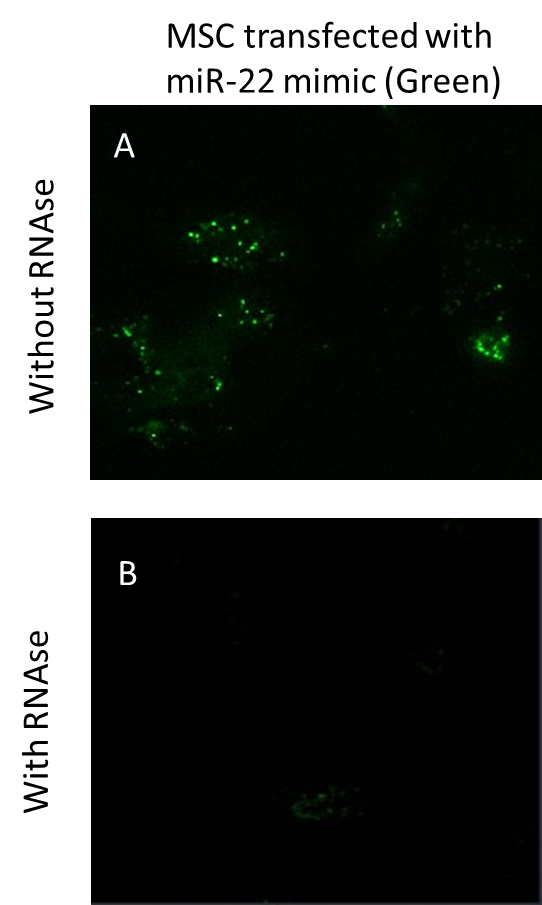


Figure. S1 **RNase treatment effectively degraded miR-22 mimic.** A. miR-22 mimic (fluorescein labeled) was transfected into cultured MSCs without RNase. B. miR-22 mimic (fluorescein labeled) was transfected into cultured MSCs with RNase.
